# Using barrier screens to characterize mosquito composition, flight activity, and abdominal status in South Lampung, Indonesia

**DOI:** 10.1186/s13071-018-3031-1

**Published:** 2018-07-31

**Authors:** Jenna R. Davidson, Supratman Sukowati, Shinta, Puji Budi Setia Asih, Din Syafruddin, Robert N. Baskin, Brandy St. Laurent, William A. Hawley, Fang Liu, Thomas R. Burkot, Frank H. Collins, Neil F. Lobo

**Affiliations:** 1 Eck Institute for Global Health, University of Notre Dame, Notre Dame, IN 46556 USA; 2 Pusat Teknologi Intervensi Kesehatan Masyarakat, Badan Litbangkes Kemenkes (Center for Public Health Intervention Technology, Ministry of Health), Jakarta, Indonesia; 3 Eijkman Institute for Molecular Biology, Jakarta, Indonesia; 4 Child Development and Survival Cluster, UNICEF, Jakarta, Indonesia; 5 James Cook University, Queensland Tropical Health Alliance, QLD, Cairns, 4870 Australia

**Keywords:** *Anopheles*, Barrier screens, Bionomics, *Culex*

## Abstract

**Background:**

Mosquito sampling methods target different aspects of mosquito behavior and are subject to trap and location specific biases. The barrier screen sampling method was developed and tested to sample free-flying, blood-fed, and host-seeking mosquitoes. During a pilot study, this method was useful in obtaining an unbiased sample of mosquitoes flying between outdoor larval habitats, and sites where blood meals were obtained. However, a relatively small number of blood-fed *Anopheles* mosquitoes were collected in Indonesia during the pilot study. The sampling method was extended in South Lampung, Indonesia, to enable the collection of blood-fed mosquitoes. This study aimed to intercept mosquitoes flying between human habitations and larval habitats with a barrier screen and to characterize mosquito composition, flight characteristics (direction, height and time), abdominal status, and parity.

**Results:**

Barrier screens intercepted 15 different mosquito species in South Lampung: eight *Anopheles* spp. and seven *Culex* spp. Species compositions varied among the villages in South Lampung. About 15% of *Anopheles* spp. caught were blood-fed, of which 28.2% of those tested had fed on humans. This is the first time human blood-fed anophelines have been collected in Indonesia using barrier screens. Blood meals identified included cow, dog, goat, and human, as well as mixed blood meals. Activity of unfed *An. subpictus*, the primary vector collected, flying towards human habitations peaked between 20:00–12:00 h, with a slow decline in activity until 18:00 h. Unfed and fed *An. sundaicus*, had a different activity profile compared to *An. subpictus*. Other species demonstrated varied peak activity times, with earlier activity occurring as a general trend. For the *Anopheles* mosquitoes collected, 55.5% were collected below 0.5 m and 83.9% were captured resting < 1 m from the ground. Parity dissections enabled age structure by species, which revealed species-specific traits such as nulliparous *An. subpictus* being more active early in the night relative to *An. sundaicus*.

**Conclusions:**

This study demonstrates that barrier screens are an effective mosquito sampling method that can be used to gain insights into local mosquito species composition, flight characteristics (direction, height and time), abdominal status, and parity.

## Background

Malaria is transmitted by *Anopheles* mosquitoes; this genus includes 465 recognized species and more unidentified members of species complexes [1]. Forty-one of these species are considered dominant malaria vectors [2, 3]. Besides being a ubiquitous biting nuisance, *Culex* mosquitoes transmit several arboviral diseases and filarial worms [4–8]. Interventions such as long-lasting insecticidal nets (LLINs) and indoor residual spraying (IRS) are often applied regardless of the local vector bionomics, even though intervention efficacy depends on mosquito behavior. For example, both LLINs and IRS reduce malaria transmission by targeting primarily indoor biting and indoor resting mosquitoes [9–11], and are therefore suboptimal intervention strategies for outdoor-biting mosquitoes. Understanding species compositions, their bionomic characteristics, and their potential susceptibility to intervention strategies is fundamental to effective disease control.

Sampling methods have limitations and biases in the context of specific behaviors of mosquitoes [12–18]. Although human landing catches (HLCs) [19] are the gold standard for trapping female, human-biting *Anopheles*, they do have several limitations. In addition to ethical concerns [20], it is impossible to use HLCs to discern the rate of human feeding without a using a second trapping method. Furthermore, HLCs only partially characterize primarily zoophilic or zoophagic species’ behaviors, which may only be captured through HLCs if a coincidental opportunistic feeding event occurs. Although efforts to develop a substitute, exposure-free trap are ongoing (e.g. the Ifakara tent trap [21, 22] and the Electric Grid [23]), none have been found to be comparable to HLCs. Moreover, none of these sampling methods (including HLCs) assess the flight direction of mosquitoes with respect to human habitation [22, 24].

There is a need for a method that efficiently samples mosquitoes outdoors while investigating flight direction. Data on chronological and spatial variances in mosquito activities, such as the peaks and bases in activity, are a prerequisite to implement appropriate interventions for the reduction of disease transmission. Further complicating the understanding of intervention efficacy, mosquito populations may exhibit behavioral resistance in response to control strategies. Behavioral resistance is defined as any alteration in behavior that aids evasion of insecticides [25, 26]. Studying behavioral changes and other adaptations in vectors [27–30] is becoming more vital with the push towards malaria elimination. Due to the lack of unbiased sampling methods for mosquitoes outdoors, the study of vector behavioral resistance remains a significant challenge for researchers.

The barrier screen sampling method was developed and evaluated successfully [14] to sample free-flying, blood-fed, and host-seeking mosquitoes outdoors. Spatial and temporal information regarding mosquito populations can be gathered with relatively limited effort - an advantage the barrier screen provides compared to other methods, which may require significant time and effort in exchange for a low rate of return and limited directional data [27]. Barrier screens provide an easy and economical way to collect mosquitoes and gather information about flight time, direction, and height. The pilot study in Indonesia, Solomon Islands, and Papua New Guinea [14] concluded that the barrier screen trapping method is sufficient to detain and allow the collection of mosquitoes, especially those with exophilic behaviors. However, the study had a limited amount of collection nights and caught few blood-fed anophelines, none of which had human blood meals in Indonesia [14].

In this study, the barrier screen sampling method was extended to four villages in South Lampung, Sumatra. Mosquitoes flying between human habitations and larval habitats were intercepted by a barrier screen and characterized for species composition, flight characteristics (direction, height and time), abdominal status, and parity. This study included both *Anopheles* and *Culex* mosquitoes. The aims of this study are to (i) further characterize *Anopheles* and *Culex* species compositions in Lampung, Indonesia; (ii) assess information about species’ abdominal status, activity time, height of activity, and flight direction as determined by barrier screens; (iii) evaluate barrier screens for use in sampling blood-fed mosquitoes outdoors in Indonesia. Lastly, this study is the first evaluation of implementing barrier screens to gather information regarding flight direction into the village from the larval habitat and out of the village towards the larval habitat.

## Methods

### Study sites

Barrier screen collections took place in four coastal villages in the Lampung District of southern Sumatra, in western Indonesia. Local industries include fishing and shrimp/fish farming. Houses are generally constructed with brick or wood and plaster, tiled roofs, and screens on some windows and eaves. This area has low to intermediate malaria endemicity that is seasonal and coincident with the rainy season (October to March). Mosquito collections took place over 39 nights (8 nights in Lempasing, 10 in Sidodadi, 2 in Hanura, and 19 in Sukaraja villages) in 2010 and 2011 (Table 1). At each study site, one 10 m long barrier screen was utilized per collection night.

**Table 1:** Barrier screen collection study sites and dates for South Lampung Province, Indonesia

District	Sub-district	Village	Collection month (no. of nights)	GPS coordinates
Lampung	Rajabasa	Sukaraja	May 2010 (1)	05°49'49.8"S, 105°36'21.6"E
			August 2010 (7)	
			October 2010 (8)	
			June 2011 (3)	
Pesawaran	Padangcermin	Hanura	March 2011 (2)	05°31'7.5"S, 105°14'31.3"E
Pesawaran	Padangcermin	Lempasing	May 2011 (4)	05°30'15.2"S, 105°15'21.1"E
			June 2011 (4)	
Pesawaran	Padangcermin	Sidodadi	January 2011 (4)	05°33'21.1"S, 105°14'29.3"E
			February 2011 (2)	
			March 2011 (4)	

### Barrier screen construction and location

Barrier screens were constructed with grey, 2 m high polyvinylchloride coated polyester netting (http://www.botexsales.com/) secured to wooden poles at 2 m intervals for a length of 10 m. Barrier screen mesh was small enough to impede the passage of a mosquito through the netting. Care was taken to minimize/eliminate spaces between the ground and the bottom of the netting [14]. Barrier screens were placed in open spaces at the edge of the village, parallel and close (10–15 m) to the vegetation outside the village. Larval habitat surveys permitted the placement of barrier screens in a direct line between the habitats and the closest village houses. Barrier screens were placed in the same position for the duration of the study at each site.

### Mosquito sampling

Barrier screens were examined for mosquitoes hourly between 18:00 h and 06:00 h. Two collectors walked down each side of the trap for 15–20 min every hour, using a flashlight to spot and mouth aspirator to collect intercepted mosquitoes [14]. The flight direction (determined by the side of the barrier screen) and height above ground (< 0.5 m; 0.5 to < 1.0 m; 1–2 m) was recorded for each mosquito. Mosquitoes were morphologically identified to species in the field [31]. Abdominal status (blood-fed, unfed, gravid and half-gravid) and sex were recorded by visual inspection. Unfed female mosquitoes were randomly selected throughout the night and dissected for parity status using the Detinova method [32]. Male mosquitoes were documented in 2010 only. *Culex* were not collected in Hanura due to the large numbers of *Anopheles* collected and limited resources to process all samples.

### Laboratory analysis

A small random sample of morphologically identified *An. sundaicus* were sequenced at the internal transcribed spacer 2 (ITS2) of the ribosomal rRNA gene [33] to confirm PCR species identifications. Abdomens of blood-fed mosquitoes were analyzed for blood meal using a diagnostic PCR assay based on vertebrate mitochondrial *cytochrome b* DNA sequences [34]. Primers were used to identify known local domestic host blood meal sources: humans, cattle, goats, dogs and pigs.

## Results

### Species composition

Mosquitoes (*n* = 6692) from eight *Anopheles* and seven *Culex* species were trapped in southern Lampung (four villages over 39 catching nights) using the barrier screen method. For *Anopheles* (*n* = 3075), the most abundant species was *An. subpictus* (78.6%). Other *Anopheles* trapped were *An. sundaicus* (9.4%), *An. vagus* (6.8%), *An. barbirostris* (3.1%) and *An. kochi* (1.6%) (Table 2). Less than 1% of the mosquitoes were *An. annularis*, *An. barbumbrosus* and *An. tessellatus*. *Anopheles* mosquito catches per barrier screen ranged from 0 to 1379 (Hanura village) per night. ITS2 sequencing revealed that 17 morphologically identified *An. sundaicus* samples sequenced were *An. epiroticus*. Despite this, the species will be referred to as *An. sundaicus*, as molecular analysis was not performed on the remaining (*n* = 272) *An. sundaicus* specimens. A large number of *Culex* mosquitoes (*n* = 3618) were caught in South Lampung (Table 2) in 3 villages (Lempasing, Sidodadi and Sukaraja). Of the seven species, *Cx. vishnui* (79.5%) and *Cx. quinquefasciatus* (19.4%) were the most common with the remaining species (*Cx. bitaeniorhyncus*, *Cx. gelidus*, *Cx. nigropunctatus*, *Cx. pallidothorax* and *Cx. tritaeniorhynchus*) comprising less than 1.2% of the *Culex* collections (Table 2).

**Table 2:** Distribution of *Anopheles* and *Culex* species over four sampling villages in South Lampung, Indonesia

Morphological species/ Locality	Lempasing (*n* = 8)		Sidodadi (*n* = 10)		Hanura (*n* = 2)		Sukaraja (*n* = 19)	
	Count	%	Count	%	Count	%	Count	%
*An. annularis*	0	0	0	0	0	0	2	1.9
*An. barbumbrosus*	0	0	2	0	0	0	0	0
*An. barbirostris*	29	17.6	11	3.9	55	2.2	0	0
*An. kochi*	15	9.1	28	10.0	4	0.2	2	1.9
*An. subpictus*	2	1.2	27	9.6	2387	94.7	0	0
*An. sundaicus*	82	49.7	141	50.2	0	0	66	62.3
*An. tessellatus*	4	2.4	0	0	6	0.2	1	0.9
*An. vagus*	33	20.0	72	25.6	68	2.7	35	33.0
*Cx. bitaeniorhyncus*	0	0	2	0.1	0	0	11	1.3
*Cx. gelidus*	3	0.5	0	0	0	0	0	0
*Cx. nigropunctatus*	0	0	6	0.5	0	0	0	0
*Cx. pallidothorax*	0	0	9	0.9	0	0	0	0
*Cx. quinquefasciatus*	0	0	71	6.8	0	0	632	76.0
*Cx. tritaeniorhynchus*	1	0.2	12	1.1	0	0	0	0
*Cx. vishuni*	565	99.2	931	90.3	0	0	188	22.6

*Abbreviation*: n, collection nights

Count was calculated as the total number of mosquitoes for each species. Percentage was calculated separately for *Anopheles* and *Culex* dividing by the overall number of mosquitoes for each study site

Both *Anopheles* and *Culex* mosquito species compositions varied from village to village within South Lampung (Table 2). The only two *Anopheles* species captured at all four locations were *An. kochi* and *An. vagus* (Table 2). Dominant species (more than ~10%) captured were *An. sundaicus* (*s.l.*) (62.3%) and *An. vagus* (33.0%) in Sukaraja; *An. subpictus* (94.7%) in Hanura; *An. sundaicus* (49.7%) and *An. barbirostris* (17.6%) in Lempasing; and *An. sundaicus* (50.2%), *An. vagus* (25.6%) and *An. kochi* (10%) in Sidodadi. Other species, captured in lower proportions (specific to villages) included *An. annularis*, *An. tessellatus* and *An. barbumbrosus. Culex* mosquitoes were collected in Sukaraja, Lempasing, and Sidodadi in South Lampung, with *Cx. quinquefasciatus* and *Cx. vishnui* collected at all three sites. *Culex bitaeniorhyncus* was captured in Sukaraja and Sidodadi, while *Cx. gelidus* was only collected in Lempasing. Six *Culex* mosquito species were collected in Sidodadi: *Cx. bitaeniorhyncus*, *Cx. nigropunctatus*, *Cx. pallidothorax*, *Cx. tritaeniorhynchus*, *Cx. quinquefasciatus* and *Cx. vishnui.*

### Bionomics

#### Flight activity and direction

**Fig. 1:**
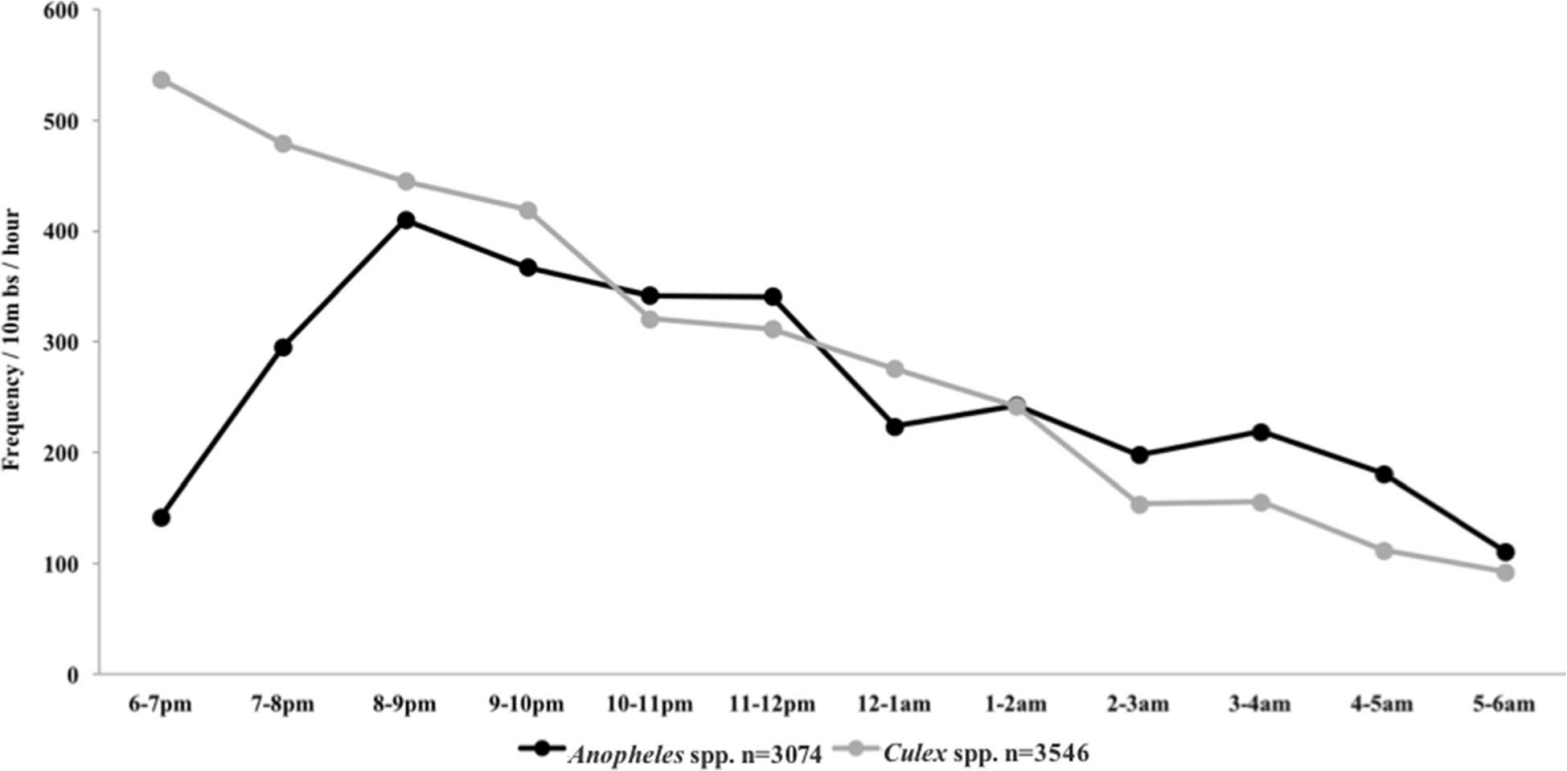
Frequency of mosquitoes by collection hour. Frequency was calculated as the number of mosquitoes resting on the 10 m barrier screen for each time point throughout the night. The number of overall *Anopheles* species peaked between 20:00–21:00 h and then steadily declined throughout the night. For *Culex* species, the major collection peak occurred between 18:00–19:00 h indicating that primary activity peaks might occur outside these collection times.

**Fig. 2:**
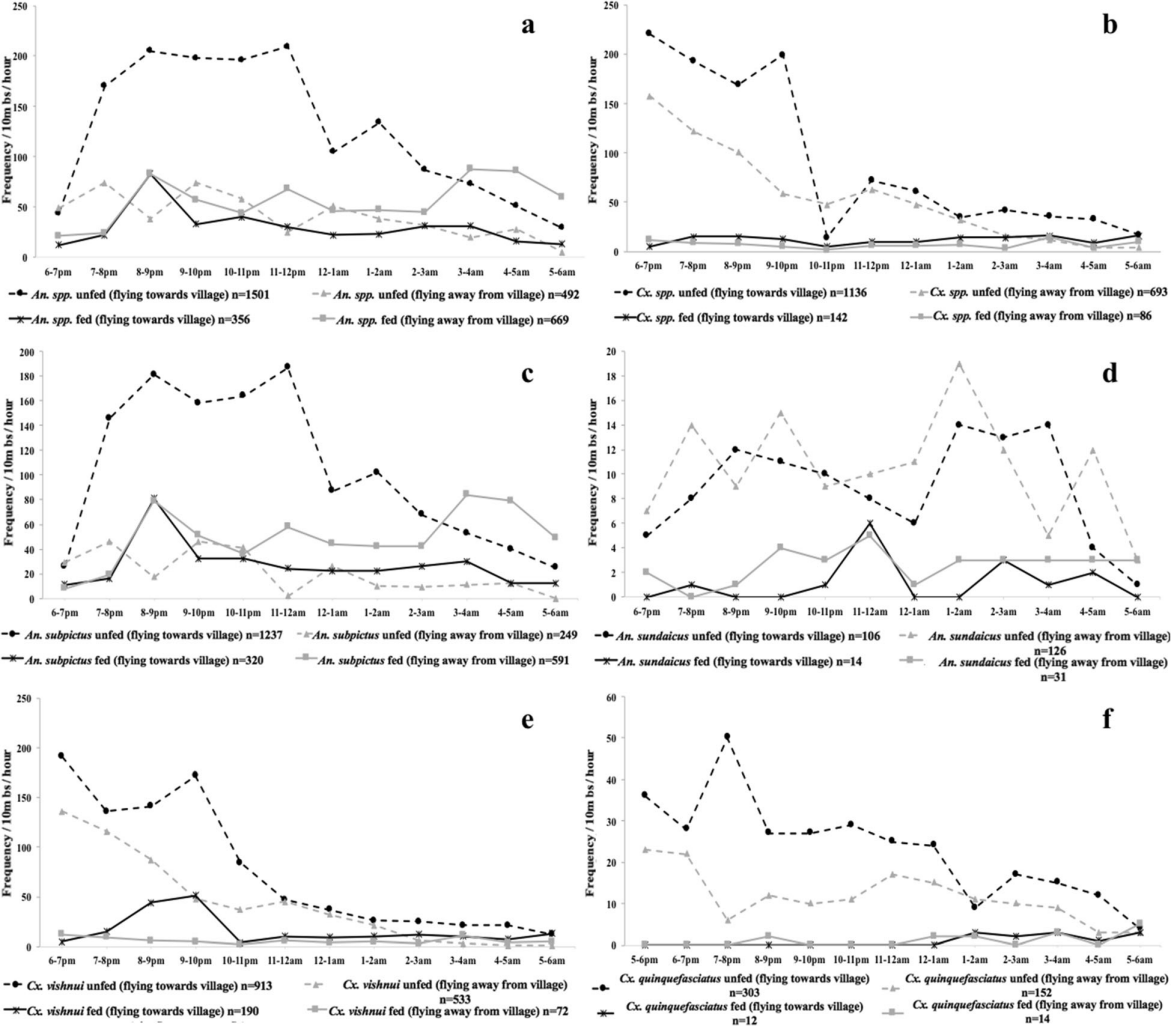
Frequency of unfed and fed mosquitoes by collection hour and direction. Frequency was calculated as the number of mosquitoes resting on the 10 m barrier screen for each time point throughout the night based on abdominal status and flight direction for the duration of the study. **a** All *Anopheles* spp. **b** *Culex* spp. **c** *An. subpictus.* **d** *An. sundaicus.* **e**. *Cx. vishnui.* **f** *Cx. quinquefasciatus*

The flight activity of *Anopheles* mosquitoes peaked between 20:00–21:00 h and then steadily declined throughout the night (Fig. 1)*.* Unfed mosquitoes flying towards the village were the largest subset of mosquitoes caught on the barrier screen (2637/5075) (Fig. 2a-f). Activity for unfed *An. subpictus* flying towards the village peaked between 20:00–12:00 h and slowly declined until 06:00 h (Fig. 2c). Approximately, five times fewer unfed *An. subpictus* were found flying away from the village (249/1237) (Fig. 2c). Although unfed *An. subpictus* were caught starting at 18:00 h, fed species members were only captured after 20:00 h (Fig. 2c). Approximately half the number of fed *An. subpictus* were seen flying towards the village (*n* = 320) relative to those caught flying away from the village (*n* = 591). Fed *An. subpictus* flying away from the village peaked in the early morning hours as unfed mosquito activity declined (Fig. 2c).

Unfed and fed *An. sundaicus*, had a different activity profile than *An. subpictus*. Approximately equal numbers of unfed mosquitoes were captured flying away from (*n* = 126) and towards (*n* = 106) the village, with slightly more found flying away from the village (Fig. 2d). Though about double the number of fed mosquitoes were captured flying away from the village in both species (Fig. 2c, d), the proportion of fed samples (relative to the total number caught for that species) was much greater in *An. subpictus* than that of *An. sundaicus* (38 *vs* 16%).

Unfed *Cx. vishnui* peaked in activity between 18:00–19:00 h and steadily declined throughout the night, with a smaller peak between 21:00–22:00 h (Fig. 2e). A peak in activity of fed *Cx. vishnui* mosquitoes flying towards the village occurred between 20:00–22:00 h (Fig. 2e). Unfed *Cx. quinquefasciatus* flying towards the village peaked in activity between 19:00–20:00 h and slowly declined throughout the night (Fig. 2f).

#### Height of capture

For *Anopheles* mosquitoes collected, 55.5% (1078/3075) were captured resting below 0.5 m from the ground. A smaller proportion 28.4% (872/3075) were resting within 0.5 m and 1 m from of the ground. The remaining 16.1% (495/3075) of *Anopheles* mosquitoes were collected resting between 1–2 m from the ground. There was no specific species that had a preferential capture height. Resting heights on the barrier screens were similarly distributed for *Culex* mosquitoes. For *Culex* mosquitoes collected, 54.1% (1959/3618) were captured resting below 0.5 m from the ground. A smaller proportion 28.2% (1020/3618) were resting within 0.5 m and 1 m from the ground. The remaining 17.7% (639/3618) of *Culex* mosquitoes were collected resting between 1–2 m from the ground. For *Anopheles* mosquitoes in which fed abdominal status was recorded, 86.4% (886/1025) were collected resting below 1 m from the ground. For *Culex* mosquitoes in which fed abdominal status was recorded, 88.6% (203/229) were collected resting below 1 m from the ground. Overall, fed mosquitoes were found lower than unfed mosquitoes.

#### Abdominal status

Of the blood-fed mosquitoes collected resting on the barrier screen, 81.7% (1025/1254) were anophelines and 18.3% (229/1254) were culicines. For the *Anopheles* mosquitoes collected on the barrier screen in which abdominal status was recorded, 33.5% (1025/3056) had blood-fed, 65.2% (1993/3056) were unfed, and the remaining 1.2% (38/3056) were either gravid or half gravid. When looking at specific species, blood-fed capture rates ranged from 8% (*n* = 17, *An. vagus*) to 41% (*n* = 39, *An. barbirostris*) of the total number caught for that species. For the *Culex* mosquitoes collected on the barrier screen in which abdominal status was recorded, 10.0% (229/2282) had blood-fed, 84.5% (*n* = 1929/2282) were unfed, and the remaining 5.4% (124/2282) were either gravid or half gravid.

#### Blood-meal identifications

A small random number of engorged females (147/1254) were tested for blood meal with PCR to identify the host animal. For *An. subpictus*, 87.5% and 4.2% of identified blood meals were on cow and human respectively (*n* = 22 successful PCR reactions, 2 could not be identified). For *An. sundaicus*, 59.1%, 13.6%, 9.1% and 4.5% of identified blood meals were on human, goat, dog, and human and goat, respectively (*n* = 19 successful PCR reactions, 3 could not be identified). *Anopheles barbirostris* fed on goat and human 83.3% and 16.7%, respectively (*n* = 6 successful PCR reactions). *Anopheles vagus* fed on goat and human 60.0% and 20.0%, respectively (*n* = 4 successful PCR reactions, 1 could not be identified). *Anopheles kochi* only fed on goat (*n* = 1 successful PCR reaction, 1 could not be identified). For *Cx. vishnui*, 47.2%, 23.6%, 13.9% and 2.8% of identified blood meals were on goat, human, dog, and goat and human, respectively (*n* = 63 successful PCR reactions, 9 could not be identified). For *Cx. quinquefasciatus*, 25.0%, 18.6% and 18.6%, of identified blood meals were on dog, goat, and human, respectively (*n* = 10 successful PCR reactions, 6 could not be identified).

#### Parity status

**Fig. 3:**
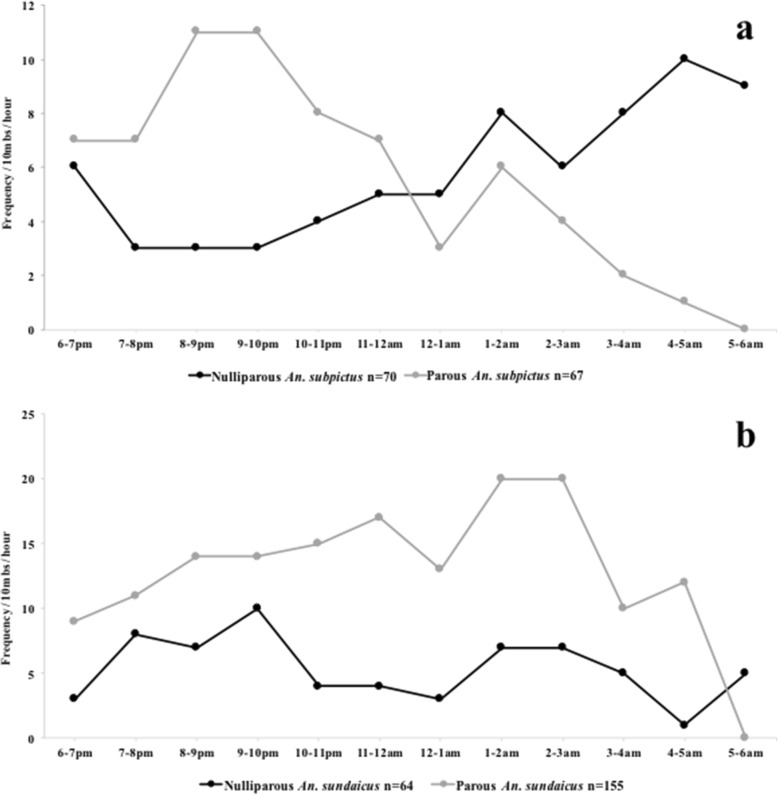
Frequency of nulliparous and parous mosquitoes by collection hour. Frequency was calculated as the total number of mosquitoes for each time point throughout the night based on parity status for (**a**) *An. subpictus* and (**b**) *An. sundaicus*

The overall parity rate for anophelines was 61.7% (356/577). For *An. subpictus* 48.9% (67/137) were parous (Table 3). However, the majority of *An. sundaicus* 70.7% were parous (155/219) (Table 3). Both species demonstrated different activity profiles with parous *An. subpictus* being more active early in the night, peaking at 20:00–22:00 h, with decreasing activity over the rest of the night (Fig. 3a). Nulliparous *An. subpictus* had increasing activity over the night peaking between 04:00–05:00 h (Fig. 3a). Parous *An. sundaicus* were consistently more active throughout the night, than nulliparous mosquitoes (Fig. 3b). The parity rate for culicines was 49.0% (292/596). Parity behavior for culicines had decreasing activity for both nulliparous and parous sets over the course of the night.

**Table 3:** Species-specific parity rates

Morphological species	Parous	Nulliparous	Parity (%)
*An. barbirostris*	41	27	60.3
*An. kochi*	28	6	82.4
*An. subpictus*	67	70	48.9
*An. sundaicus*	155	64	70.8
*An. vagus*	59	48	55.1
*Cx. quinquefasciatus*	52	50	60.0
*Cx. vishuni*	228	246	48.1

Parity was calculated as the number of parous mosquitoes for each species. Parity rates for species with less than 30 specimens are not reported

A single male *An. sundaicus* specimen was caught in 2010 (the only year when males were documented). However, *Cx. quinquefasciatus* (*n* = 160) as well as *Cx. vishnui* males (*n* = 74) were trapped, comprising 6% of the total *Culex* captured.

## Discussion

Identifying local mosquito vector compositions and their bionomic traits is a vital step in comprehending disease transmission dynamics. Towards understanding outdoor mosquito behaviors and bionomic traits, the barrier screen [14] was implemented at four sites in Lampung, Indonesia.

During this evaluation of the barrier screen, 15 species of mosquitoes were captured, including eight additional species not captured during the pilot evaluation [14]. These additional species include both *Anopheles* and *Culex* species: *An. barbumbrosus*, *An. barbirostris*, *An. subpictus*, *Cx. bitaeniorhyncus*, *Cx. gelidus*, *Cx. nigropunctatus*, *Cx. pallidothorax* and *Cx. tritaeniorhynchus* (Table 2).

Species compositions varied widely between close geographical areas. Though some species were present in multiple sampling sites (*An. kochi*, *An. subpictus, An. vagus*), each study site had unique vector composition and density. These differences are attributed to the presence of available larval habitats preferred by the species: *An. subpictus* prefers more inland, freshwater sites, while *An. sundaicus* often exploits slightly saline habitats created by fish farming and streams linked to coastal sea water [35]. Similar site-specific differences were seen with *Culex* mosquitoes. The variation in mosquito species and population densities between closely located (< 20 km apart) villages in South Lampung demonstrates that local vector compositions, and consequently, characteristics of disease transmission, may have substantial variations on a small geographical scale. Meanwhile, of the eight *Anopheles* species captured in this study, seven are described primary vectors in Indonesia [36–44]. Similarly, of the seven *Culex* species captured, five are described primary vectors of arboviral diseases and/or filariasis [45–48]. The diversity of primary disease vectors in Indonesia highlights the importance of continued and expanded sampling methodology.

The barrier screen can be used to intercept free-flying mosquitoes outdoors, making it a useful tool to evaluate trap-specific biases. In South Lampung, unfed *An. subpictus* flying towards the village peaked during the first half of the night. Similar peak flight times have been reported from the Lesser Sundas and Sulawesi [49]. However, in other regions of Indonesia, *An. subpictus* flight activity peaks during the second half of the night [50]. Both of these studies utilized HLCs, indoor-resting collections, and animal baited tent traps to complete their collections [49, 50]. In this study, *An. sundaicus* activity peaked between 02:00–03:00 h. This finding differs from literature published about *An. sundaicus* in Western Java, which indicated high biting activity during the first and last quarters of the night [51]. However, this study aligns with literature published from Central Java, which found *An. sundaicus* feeding activity to peak during the second and third quarters of the evening [39, 49]. One explanation for these discrepancies is that local mosquito species’ peak flight times may differ when evaluated using different sampling methods. The barrier screen’s ability to intercept free-flying mosquitoes may also indicate trap specific biases in data from other traps, like HLCs and animal baited traps when they are used to determine mosquito activity. Additionally, these discrepancies in published literature may be due to changes in behavior, site-specific differences, or species-specific differences, as a randomly selected subsample of *An. sundaicus* was molecularly identified to *An. epiroticus.*

The barrier screen reveals preliminary data that suggests mosquito host-seeking and resting behaviors. As expected, generally more unfed mosquitoes than fed mosquitoes were collected on the barrier screen. It can be hypothesized that an unfed mosquito may fly directly towards the village for a blood meal from a larval habitat (the barrier screen was placed in a direct line between the two), while a fed mosquito may fly in any direction out of the village, rest inside houses, or rest within the village, avoiding the single barrier screen. There were more unfed female *Anopheles* mosquitoes flying towards the village than flying away. Meanwhile, there were more fed *Anopheles* mosquitoes captured flying away from the village than flying towards, and the peaks of fed *Anopheles* mosquitoes always followed unfed activity peaks. While this suggests that unfed female mosquitoes trapped on the outside of the barrier screen (flying towards human habitation) are doing so to obtain blood meals, further studies would have to investigate the strength of this relationship.

Both *An. subpictus* and *An. sundaicus* had varying rates of capture relative to abdominal status. This may indicate longer resting rates for *An. subpictus* and delayed activity times for *An. sundaicus*. For example, unfed *An. subpictus* flying towards village peaked between 20:00–21:00 h, which was not followed by a fed activity peak flying away from the village until 03:00–04:00 h. This may indicate that *An. subpictus* rests in the village immediately after feeding, before flying away from human habitation. Meanwhile, unfed *An. sundaicus* flying towards the village peaked at 20:00–21:00 h, immediately followed by fed *An. sundaicus* flying away from the village peaking at 21:00–22:00 h. This suggests that *An. sundaicus* may return directly to the larval habitat after feeding in the village (without resting). Additionally, unfed *An. sundaicus,* flying toward the village had two early morning peaks at 01:00–02:00 h and 03:00–04:00 h, suggesting delayed activity times. However, these findings may also point to sampling biases with this method. Additional collections with associated indoor and outdoor village resting collections, may enable an evaluation of the barrier screen’s ability to measure these resting behaviors.

This study corroborates the claim that host-seeking species primarily fly at levels of a meter or less above the ground. The height at which mosquitoes were caught was evaluated towards understanding how flight height may affect barrier screen sampling. Previous data [52, 53] demonstrated that most mosquitoes fly close to the ground when foraging. This was seen during this study as well, for both *Culex* and *Anopheles* samples, with no distinction for any single species. Supporting reports that many host-seeking species fly primarily at levels of a meter or less above the ground, 83.9% and 82.3% of the *Anopheles* and *Culex* captured were below 1 m.

The barrier screen impartially captures blood-fed, free-flying mosquitoes outdoors. Other sampling methods, such as pyrethroid spray catches, indoor aspirations, and the CDC-light trap introduce location or host biases when sampling blood-fed mosquitoes. In this study, the barrier screen captured large numbers of blood-fed mosquitoes. Overall, 34% of the *Anopheles* and 10% of the *Culex* samples were blood-fed. The analysis of unbiased blood meal samples enables accurate inferences on host preferences as well as changes in population wide behaviors over time.

This is the first time human blood-fed anophelines have been collected in Indonesia using barrier screens. This may indicate that *An. sundaicus* and *An. vagus* are more opportunistic feeders than previously believed. This small set of results is encouraging: indicating that the barrier screens, with proper positioning, may be useful in obtaining zoophilic, anthropophagic, and opportunistic blood-fed mosquitoes.

This study used parity analysis to determine the age structure of mosquito field populations: an important determinant of vectorial capacity [32]. Besides parity rates, parity analysis demonstrated species specific behavioral differences and periods of time when parous (older) mosquitoes were more active. The discrepancy in parous and nulliparous activity between *An. subpictus* and *An. sundaicus* demonstrates that interventions targeting overall *Anopheles* activity rates may not be targeting the higher-risk, parous, subset of mosquitoes. Future studies connecting age structures of local vector populations to disease transmission times could reveal that intervention strategies that target overall peak times for a species do not appropriately address disease transmission risks from parous populations.

Additional collections and analyses were done using barrier screens in Seram and Papua, Indonesia. However, due to limited sample sizes, the datasets are not shown. The studies at both these sites reflected similar use of the barrier screen to collect information on vector species and their flight behaviors. The barrier screens caught more or equal number of mosquitoes when compared to HLCs in Seram (data not shown due to small sample size). The barrier screen was used to sample and characterize mosquito behaviors in eastern Indonesia (Lampung), western (Papua) as well as more central (Seram), which represent Asian and Australian fauna.

The ability of barrier screens to capture free-flying mosquitoes that encounter and rest on them, irrespective of indoor, outdoor, temporal, or host preferential behaviors is dependent on proper placement and orientation [14]. Limitations of barrier screens include their inability to capture mosquitoes that do not venture into their direct path. In this case, this would include the populations of mosquitoes that do not enter villages to feed, those that fly higher than the barrier screen (> 2 m), those that are intercepted by the barrier screen but crawl over it before collections, and those that have alternative flight paths into the village. Future studies could include barrier screens higher than 2 m, barrier screens used in forest/oviposition/larval habitats, and barrier screens with covers to reduce or eliminate the possibility of a mosquito escaping over the screen. Benefits of barrier screens include shorter collection times compared to searching vegetation for resting mosquitoes and the ability to trap large numbers of mosquitoes per night [14, 28, 54], including blood-fed mosquitoes. Additionally, barrier screens are an economical collection strategy for remote locations and easily implemented in the field. Finally, this evaluation of the barrier screen sampling method could be helpful for improving and developing new trapping systems that account for changes in behavior as a response to interventions, while including sampling capabilities like flight direction, preferential hosts, and peak activity.

## Conclusion

Barrier screens capture free-flying mosquitoes that encounter and rest on them, irrespective of indoor, outdoor, temporal, or host preferential behaviors. This study demonstrates that barrier screens can be used to gain insights into mosquito species composition, flight characteristics (direction, height, and time), abdominal status, and parity.

## References

[1] Harbach RE. Genus *Anopheles* Meigen, 1818. Mosquito Taxonomic Inventory; 2011. http://mosquito-taxonomic-inventory.info/node/11358#.

[2] Hay SI, Sinka ME, Okara RM, Kabaria CW, Mbithi PM, Tago CC, et al. Developing global maps of the dominant *Anopheles* vectors of human malaria. PLoS Med. 2010;7:e1000209. 10.1371/journal.pmed.1000209 PMC2817710 20161718

[3] Gilles HM, Warrell DA, Service MW (1993). The *Anopheles* vector. Bruce-Chwatt’s Essential Malariology.

[4] Turell MJ (2012). Members of the *Culex pipiens* complex as vectors of viruses. J Am Mosq Control Assoc..

[5] Converse JD, Tan RI, Rachman IT, Lee VH, Shope RE (1985). Ingwavuma virus (Simbu group) from *Culex* and *Mansonia* mosquitoes (Diptera: Culicidae) in Indonesia. J Med Entomol..

[6] Murty US, SatyaKumar DV, Sriram K, Rao KM, Singh TG, Arunachalam N (2002). Seasonal prevalence of *Culex vishnui* subgroup, the major vectors of Japanese encephalitis virus in an endemic district of Andhra Pradesh, India. J Am Mosq Control Assoc..

[7] Sudeep AB (2014). *Culex gelidus*: an emerging mosquito vector with potential to transmit multiple virus infections. J Vector Borne Dis..

[8] Manimegalai K, Sukanya S (2014). Biology of the filarial vector, *Culex quinquefasciatus* (Diptera: Culicidae). Int J Curr Microbiol App Sci..

[9] Killeen GF (2014). Characterizing, controlling and eliminating residual malaria transmission. Malar J..

[10] Enayati A, Hemingway J (2010). Malaria management: past, present, and future. Annu Rev Entomol..

[11] Bugoro H, Cooper R, Butafa C, Iro’ofa C, Mackenzie D, Chen C-C, et al. Bionomics of the malaria vector *Anopheles farauti* in Temotu Province, Solomon Islands: issues for malaria elimination. Malar J. 2011;10:133. 10.1186/1475-2875-10-133 PMC3123245 21592366

[12] Rohani A, Zamree I, Ali WNWM, Hadi AA, Asmad M, Lubim D (2013). Nocturnal man biting habits of mosquito species in Serian, Sarawak, Malaysia. Adv Entomol..

[13] St. Laurent B, PBS A, Bretz D, Mueller J, Miller HC, Baharuddin A (2016). Behaviour and molecular identification of *Anopheles* malaria vectors in Jayapura District, Papua Province, Indonesia. Malar J..

[14] Burkot TR, Russell TL, Reimer LJ, Bugoro H, Beebe NW, Cooper RD (2013). Barrier screens: a method to sample blood-fed and host-seeking exophilic mosquitoes. Malar J..

[15] Govella NJ, Chaki PP, Killeen GF (2013). Entomological surveillance of behavioural resilience and resistance in residual malaria vector populations. Malar J..

[16] Clements AN (1999). The biology of mosquitoes.

[17] Service M (1977). A critical review of procedures for sampling populations of adult mosquitoes. Bull Entomol Res..

[18] Service MW (1977). The need for improved methods for sampling mosquito populations. Wiad Parazytol..

[19] Gimnig JE, Walker ED, Otieno P, Kosgei J, Olang G, Ombok M (2013). Incidence of malaria among mosquito collectors conducting human landing catches in western Kenya. Am J Trop Med Hyg..

[20] Achee NL, Youngblood L, Bangs MJ, Lavery JV, James S (2015). Considerations for the use of human participants in vector biology research: a tool for investigators and regulators. Vector Borne Zoonotic Dis..

[21] Sikulu M, Govella NJ, Ogoma SB, Mpangile J, Kambi SH, Kannady K (2009). Comparative evaluation of the Ifakara tent trap-B, the standardized resting boxes and the human landing catch for sampling malaria vectors and other mosquitoes in urban Dar es Salaam, Tanzania. Malar J..

[22] Govella NJ, Chaki PP, Geissbuhler Y, Kannady K, Okumu F, Charlwood JD (2009). A new tent trap for sampling exophagic and endophagic members of the *Anopheles gambiae* complex. Malar J..

[23] Majambere S, Massue DJ, Mlacha Y, Govella NJ, Magesa SM, Killeen GF (2013). Advantages and limitations of commercially available electrocuting grids for studying mosquito behaviour. Parasit Vectors..

[24] Maliti DV, Govella NJ, Killeen GF, Mirzai N, Johnson PC, Kreppel K (2015). Development and evaluation of mosquito-electrocuting traps as alternatives to the human landing catch technique for sampling host-seeking malaria vectors. Malar J..

[25] Gatton ML, Chitnis N, Churcher T, Donnelly MJ, Ghani AC, Godfray HC, et al. The importance of mosquito behavioural adaptations to malaria control in Africa. Evolution. 2013;67:1218–30. 10.1111/evo.12063 PMC3655544 23550770

[26] Gould F (1984). Role of behaviour in the evolution of insect adaptation to insecticides and resistant host plants. Bull ESA..

[27] Russell TL, Beebe NW, Bugoro H, Apairamo A, Collins FH, Cooper RD (2016). *Anopheles farauti* is a homogeneous population that blood feeds early and outdoors in the Solomon Islands. Malar J..

[28] Ndiath MO, Mazenot C, Gaye A, Konate L, Bouganali C, Faye O (2011). Methods to collect *Anopheles* mosquitoes and evaluate malaria transmission: a comparative study in two villages in Senegal. Malar J..

[29] Moiroux N, Gomez MB, Pennetier C, Elanga E, Djenontin A, Chandre F (2012). Changes in *Anopheles funestus* biting behavior following universal coverage of long-lasting insecticidal nets in Benin. J Infect Dis..

[30] Reddy MR, Overgaard HJ, Abaga S, Reddy VP, Caccone A, Kiszewski AE (2011). Outdoor host seeking behaviour of *Anopheles gambiae* mosquitoes following initiation of malaria vector control on Bioko Island, Equatorial Guinea. Malar J..

[31] O’Connor C, Soepanta A. Illustrated Key to Female Anophelines of Indonesia. Jakarta: Directorate of Communicable Disease, MoH and US Naval Medical Research; 1989.

[32] Detinova TS (1962). Age-grouping methods in Diptera of medical importance with special reference to some vectors of malaria. Monogr Ser World Health Organ..

[33] Beebe NW, Saul A (1995). Discrimination of all members of the *Anopheles punctulatus* complex by polymerase chain reaction-restriction fragment length polymorphism analysis. Am J Trop Med Hyg..

[34] Kent RJ, Norris DE (2005). Identification of mammalian blood meals in mosquitoes by a multiplexed polymerase chain reaction targeting cytochrome B. Am J Trop Med Hyg..

[35] Dusfour I, Harbach RE, Manguin S (2004). Bionomics and systematics of the oriental *Anopheles sundaicus* complex in relation to malaria tranmission and vector control. Am J Trop Med Hyg..

[36] Chandra G, Bhattacharjee I, Chatterjee S (2010). A review on *Anopheles subpictus* Grassi *-* a biological vector. Acta Trop..

[37] Elyazar IR, Sinka ME, Gething PW, Tarmidzi SN, Surya A, Kusriastuti R (2013). The distribution and bionomics of *Anopheles* malaria vector mosquitoes in Indonesia. Adv Parasitol..

[38] Saeung A (2012). *Anopheles* (Diptera: Culicidae) species complex in Thailand: identification, distribution, bionomics, and malaria-vector importance. Int J Parasitol Res..

[39] Sundararaman S, Soeroto RM, Siran M (1957). Vectors of malaria in Mid-Java. Indian J Malariol..

[40] Boewono DT. Verification of malaria vectors in Teluk Dalam Subdistrict, Nias Island. Majalah Parasitol Indonesia. 1997;10:23–32.

[41] Van Hell JC (1952). The Anopheline fauna and malaria vectors in South Celebes. Doc Neerl Indones Morbis Trop..

[42] Sinka ME, Bangs MJ, Manguin S, Chareonviriyaphap T, Patil AP, Temperley WH (2011). The dominant *Anopheles* vectors of human malaria in the Asia-Pacific region: occurrence data, distribution maps and bionomic precis. Parasit Vectors..

[43] Singh RK, Haq S, Kumar G, Dhiman RC (2013). Bionomics and vectorial capacity of *Anopheles annularis* with special reference to India: a review. J Commun Dis..

[44] Metselaar D (1956). A pilot project of residual-insecticide spraying to control malaria transmitted by the *Anopheles punctulatus* group in Netherlands New Guinea. Am J Trop Med Hyg..

[45] Carpenter SJ, Lacasse WJ (1955). Mosquitoes of North America (north of Mexico).

[46] Bram RA (1967). Contributions to the mosquito fauna of Southeast Asia. II. The genus *Culex* in Thailand (Diptera: Culiciade). Contrib Am Entomol Inst..

[47] Harbach RE (1988). The mosquitoes of the subgenus *Culex* in southwestern Asia and Egypt (Diptera: Culicidae). Contrib Am Entomol Inst..

[48] Sirivanakam S (1976). Medical entomology studies-III. A revision of the subgenus *Culex* in the Oriental Region (Diptera: Culicidae). Contrib Am Entomol Inst..

[49] Collins RT, Jung RK, Anoez H, Sutrisno RH, Putut D. A study of the coastal malaria vectors, *Anopheles sundaicus* (Rodenwaldt) and *Anopheles subpictus grassi*, in South Sulawesi, Sulawesi, Indonesia. Geneva: World Health Organization. WHO/MAL/79.913WHO/VBC/79.740

[50] Hoedojo. Bionomic *Anopheles subpictus*, specifically regarding its role as a malaria vector in Jengkalang, Flores. Majalah Parasitol Indonesia. 1992;5:47–56.

[51] Stoops CA, Rusmiarto S, Susapto D, Munif A, Andris H, Barbara KA (2009). Bionomics of *Anopheles* spp*.* (Diptera: Culicidae) in a malaria endemic region of Sukabumi, West Java, Indonesia. J Vector Ecol..

[52] Snow WF (1977). The height and direction of mosquitoes in West African savanna, in relation to wind speed and direction. Bull Entomol Res..

[53] Snow WF (1979). The vertical distribution of flying mosquitoes (Diptera: Culicidae) near an area of irrigated rice-fields in The Gambia. Bull Entomol Res..

[54] Pombi M, Guelbeogo WM, Kreppel K, Calzetta M, Traore A, Sanou A (2014). The Sticky Resting Box, a new tool for studying resting behaviour of Afrotropical malaria vectors. Parasit Vectors..

